# Integrated Evaluation of Urban Development Suitability Based on Remote Sensing and GIS Techniques – A Case Study in Jingjinji Area, China

**DOI:** 10.3390/s8095975

**Published:** 2008-09-25

**Authors:** Jiang Dong, Dafang Zhuang, Xinliang Xu, Lei Ying

**Affiliations:** State Key Lab of Resources and Environmental Information System, Institute of Geographical Sciences and Natural Resources Research, Chinese Academy of Sciences, Beijing 100101, China

**Keywords:** Urban development, Jingjinji area, Remote sensing, GIS

## Abstract

Jingjinji area (namely Beijing, Tianjin and He Bei Province) is one of the three largest regional economic communities in China. Urban expansion has sped up in the past 20 years in this area due to the rapid economic and population growth. Evaluating the land-use suitability for urban growth on a regional scale is an urgent need, because the most suitable areas and the most suitable scale of urban growth can thus be determined accordingly. In order to meet this requirement, remote sensing and geographic information system (GIS) techniques were adopted, and an integrated evaluating model was developed supported by AHP method. The integrated urban development suitability index (UDSI) was calculated using this model. According to the UDSI result, the spatial distribution of urban development suitability and its driving forces were analyzed. Urban boundaries in 1995, 2000 and 2005, which were derived from Landsat TM/ETM+ satellite data, were overlaid on the UDSI map, and the suitable urban develop tendency in this area were discussed. The result of this study indicated that integrated evaluation of urban development could be conducted in an operational way using remote sensing data, GIS spatial analysis technique and AHP modeling method.

## 1. Introduction

Chinese cities have experienced rapid population growth and continuous expansion in the past 20 years due to accelerated economic development [1]. Rapid urbanization has imposed significant pressure on environmental and natural resources [2]. It is reported that Chinese urban land increased by 8,170 km^2^ from 1990 to 2000 [3] and more attention has been paid to urban development monitoring in China [4-5]. In coming years, rapid urban increases are expected to place an increased burden on urban land and water resources, especially in areas with high rates of economic growth [6].

Jingjinji area (namely Beijing, Tianjin and Hebei Province) is one of the three large regional economic communities in China. There is a sharp conflict between urban growth and limited land/water resources. Many studies concerning urban development management were conducted in this area, and most of them focused on monitoring urban sprawl, resources carrying capacity calculations or regional resource conservation problems [7-8]. However, there is still a lack of integrated urban land-use planning for this area, leading to many socio-economic and eco-environmental consequences, such as disorganized urban development, unreasonable allocation of land-use types, and incomplete civil and environmental infrastructure [9].

Evaluation of urban development suitability plays a fundamental role in regional urban land-use planning. Its major objective is to evaluate the advantages and disadvantages for urban development certain areas, so as to find out places which are most suitable for urban development in the future [10]. In the field of suitability assessment for urban development, GIS, remote sensing and numerical modeling techniques have been proved to be efficient tools by recent studies. Collins *et al.* reviewed the land-use suitability analysis based on GIS in the United States [11]. Dai, F.C. *et al.* presented a GIS-based geo-environmental evaluation for urban land-use planning [10]. Liu, Y. *et al.* built up an integrated GIS-based analysis system for land-use management of lake area in urban fringe in central China, and Analytic hierarchy process (AHP) method was adopted to derive weights for the evaluating model [9]. GIS and AHP were also used together for land suitability analysis for urban development in the studies conducted by Aly, M.H. *et al.* [12], Mohammad A.M. *et al.* [13] and Li, A. *et al.* [14]. In general, recent studies indicated that combing use of these technologies can present a platform to support multi-level and hierarchical integrated analysis on human activities, resources and environment [14].

In the early 2007, funded by the Jingjinji Urban Planning Project, a study of integrated evaluation of the suitability for urban growth has been conducted to support the decision making of the most suitable scale and direction of urban growth. The goal of this paper is to demonstrate the methods and the main findings of this study.

## 2. Study area

**Figure 1.**
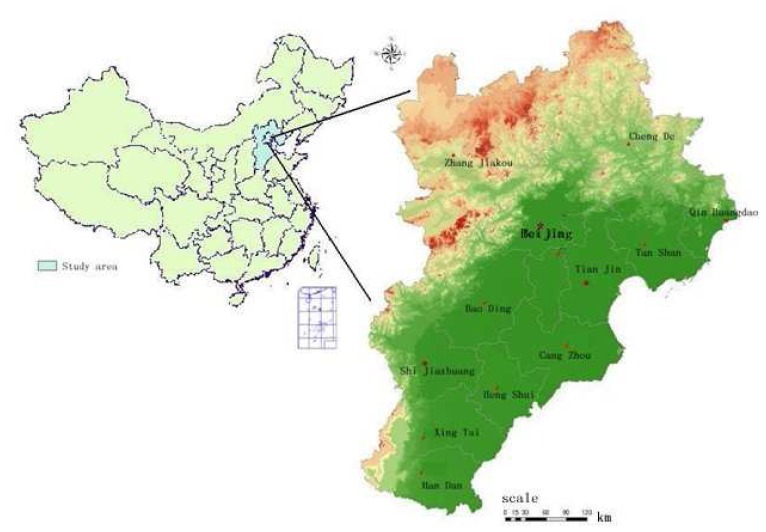
Location of the study area: Jingjinji area.

Jingjinji area, which is sited in the eastern part of the North China Plain was selected as the study area in this paper. It is located between latitudes 26°02′N and 42°38′N, and longitudes 113°25′E and 119°51′E (Figure 1). The area consists of Beijing city, Tianjin city and Hebei province, and has a total area of 213,600 km^2^ and a total population of 94.32 million. Beijing is the capital city of China with a area of 16,300 km^2^ and a population of 15.56 million in 2005. Tianjin is a municipality direct under the Central Government, as well as an expanding city. It is one of the largest cities in North China with an area of 11,700 km^2^ and a population of 10.36 million in 2005. He Bei province, with an area of 185,600 km^2^, contains 11 main cities and the total population was 68.49 million in 2005. The urbanization rate of He Bei province is 37.70% (2005), while Beijing and Tianjin have much higher levels of urbanization (83.62% and 72.11%, respectively) compared to the national average of 42.99% (2005). The Jingjinji area's GDP has been increasing by an annual rate of 11.4% since 1995.

## 3. Methodology

### 3.1 Evaluation mechanism and factors selection

Regional urban development is influenced by both natural and social-economic conditions [9]. Wu has presented the base criteria for urban land suitability assessment [18]. We adapted these criteria according to the qualitative analysis of the urbanization in Jingjinji area, together with suggestions from local experts on urban development planning, land resources, ecology, etc. An integrated evaluation criteria system was set up containing 9 factors belong to 3 categories: (1) environmental background factors, including elevation, slope, geomorphological types, accumulated temperature and wetness index; (2) water/land resources, including Precipitation, river density, land use; (3) socio-economic, including railway density, road density, and population density (Table2).

### 3.2 Data acquisition

**Figure 2.**
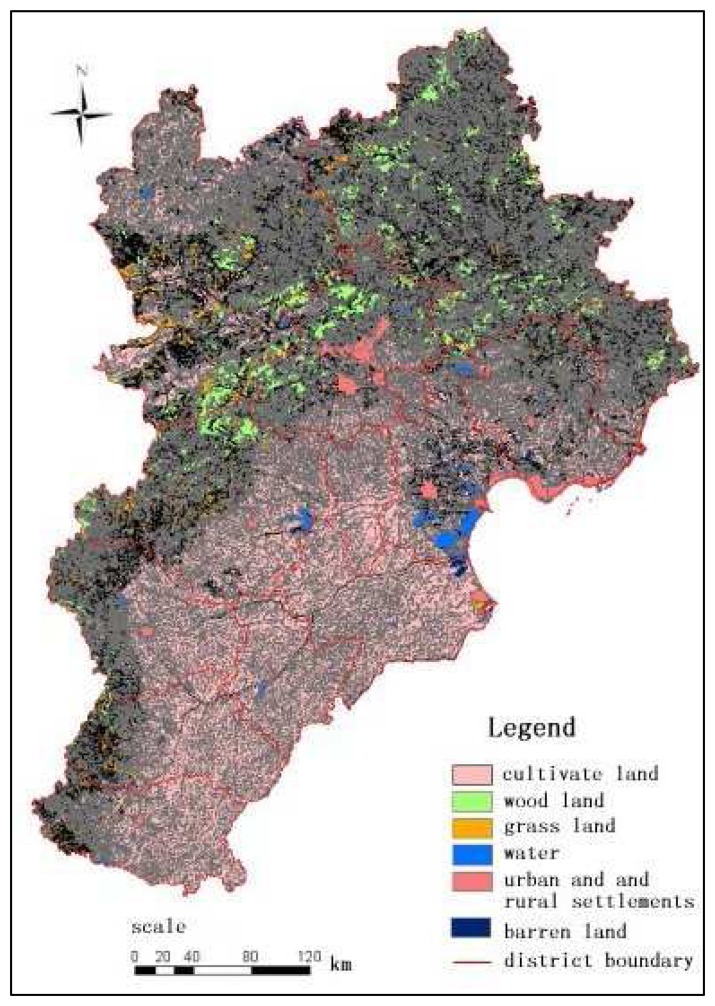
Land use pattern of Jingjinji area in 2005.

According to the evaluation criteria system mentioned above, the input data for this study include:

1) Land use data: Land use data are the most fundamental data of the evaluation model for urban development suitability and their can be derived from remote sensing images. Land use data for 2005 were obtained from Landsat TM data through human-computer interactive interpretation. Six land use types were identified including: (1) cultivated land; (2) woodland; (3) grassland; (4) water; (5) urban and rural settlements; (6) barren land. A set of ground data from field surveys were selected and used to guarantee the land use classification on accuracy (Figure 2). Meanwhile, the ‘river density’ data, which refers to the length of the river per unit area within certain statistical unit, were derived from the land use data.
2) DEM data (including elevation and slope), geomorphological types data and transportation data were supplied by State Bureau of Surveying and Cartography (SBSC)
3) Meteorological data, including annual average precipitation data, accumulated air temperature data and wetness index data, were derived from National Resources and Environmental Database presented by Resources and Environmental Scientific Data Center (RESDC), Chinese Academy of Sciences (CAS). The accumulated air temperature refers to the annual daily mean air temperature above 0°C. Wetness index is defined as actual evapotranspiration dividing by potential evapotranspiration, which were calculated from meteorological data annually.
4) Population data: 100 m × 100m grid population data were presented by RESDC, CAS. These gridded population data were transformed from census data based on the spatialization model [19].
5) Railway density and road density data were also presented by RESDC, CAS. Road density is calculated by summing the length of roads of different classes and dividing by the area of 100 m grid cell statistical unit. Two road classes were selected for calculating: national roads and highways.

Before further processing, all of the source data were re-sampled onto a raster dataset with 100 m spatial resolution. Meanwhile, the data were transformed to the same coordinate system, i.e. Albers Equal Area projection system with original longitude 105°E, double standard parallel of 27°N and 45°N, Beijing 1954 geodetic datum and Krassovsky ellipsoid.

### 3.3 Establishment of the evaluation model

**Figure 3.**
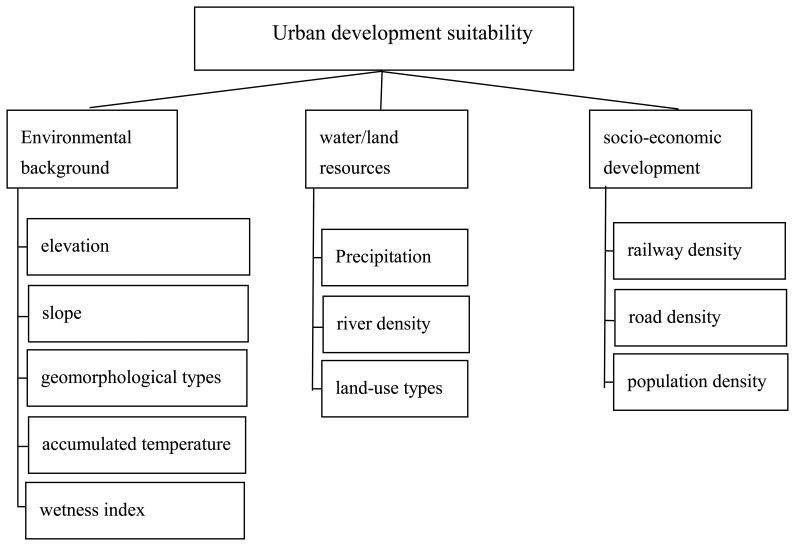
Hierarchical structure of the suitability factors for urban development in Jingjinji area

For each parameter in Table 2, a set of relative weights should be developed for urban development suitability evaluation as the next step. The analytical hierarchy process (AHP) method was adopted to derive weights assigned to the parameters. The AHP is a multi-criteria decision making method that employs a pair-wise comparison procedure to arrive at a scale of preference among a set of alternatives [9]. AHP has become a popular tool for problems of multi-attribute decision making modeling. The main steps for deriving the relative weight of parameters for urban development suitability evaluation include[20-21]:

1) Establish a criteria system for urban development suitability evaluation (as mentioned in section 3.1) and arrange these parameters in a hierarchical order (Figure 3).
2) Conduct the pair-wise comparison procedure and establish the weighting matrix. The relative importances of the selected parameters were achieved by consulting and surveying the opinions of 15 experts in the Jingjinji area. The results of this procedure are shown in Table 3.
3) Standardize the values of the parameters. Transform the original values of the parameters into relative scores. The standards for transformation are shown in Table 4.

Then, an integrated urban development suitability index (UDSI) could be calculated by using the following formula:
(1)
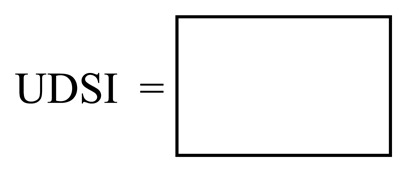
where, Wi is the weight of parameter i; C_i_ is the standardized relative score of parameter i; n is total number of parameter related to urban development suitability evaluation. The area with a higher UDSI indicates it is more suitable for urban development.

## 4. Results and analysis

### 4.1 Evaluation of suitability for urban development in Jingjinji area

**Figure 4.**
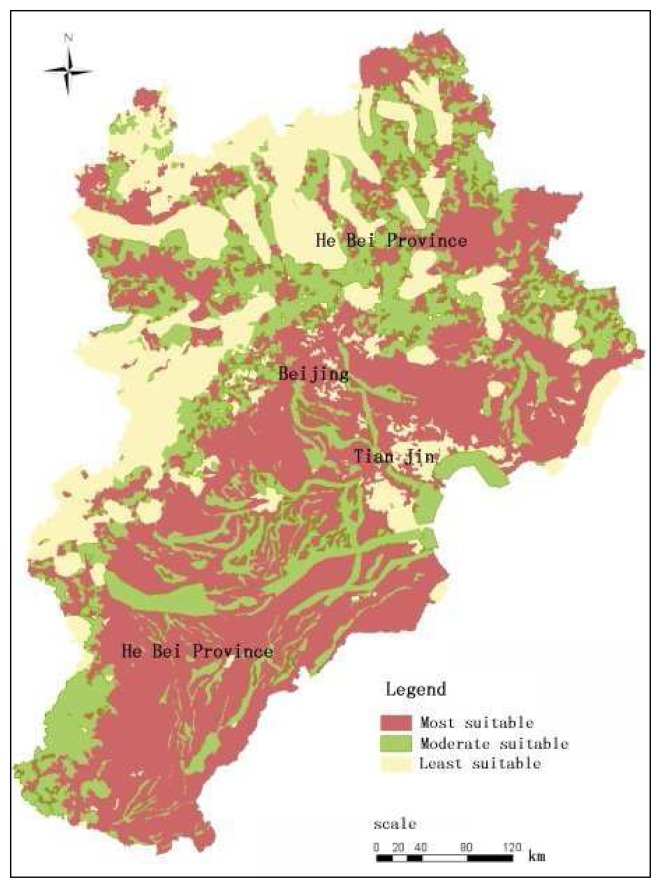
Spatial distribution of suitability for urban development in Jingjinji area.

Using data in Table 3 as inputs, the USDI of each 100 m × 100 m grid cell was calculated from formula 1. Pixels with higher USDI indicates that it is more suitable for urban development. The USDI data of Jingjinji area were grouped into three levels: (1) most suitable; (2) moderately suitable; (3) least suitable (Figure 4).

The spatial statistic result of the USDI data of Jingjinji area shows that out of the total area of 21.36 km^2^, 22.64% (4.84 km^2^) are most suitable for urban development, 30.56% (6.53 km^2^) are moderately suitable for urban development and 46.81%(10.00 km^2^) area least suitable for urban development.

### 4.2 Combination analysis of urban growth tendency and USDI map in Jingjinji area

**Figure 5.**
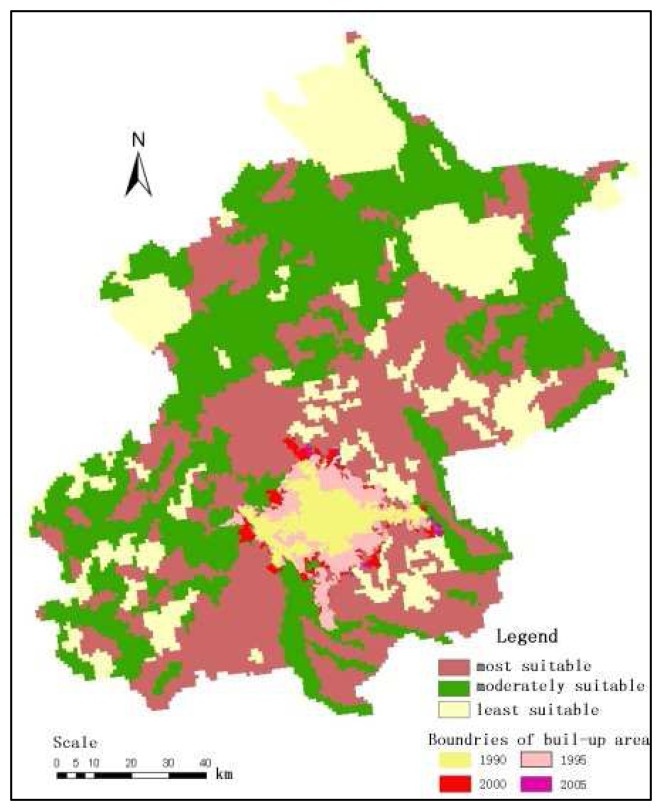
USDI map and urban expansion of Beijing city since 1990.

**Figure 6.**
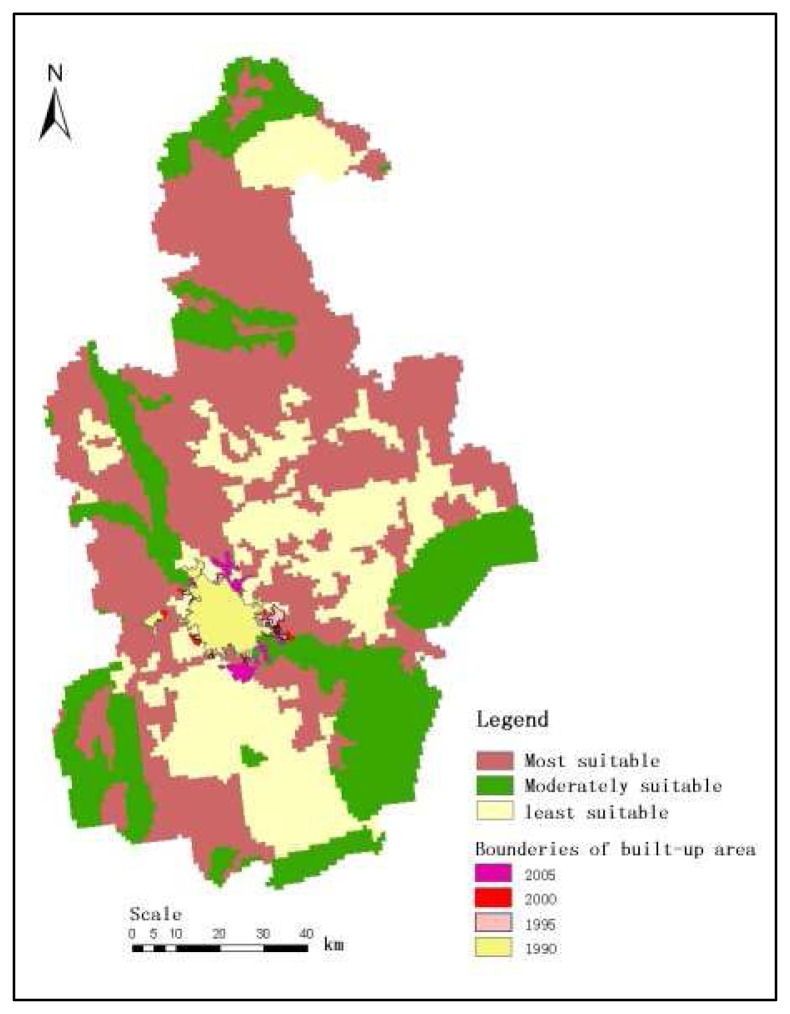
USDI map and urban expansion of Tianjin city since 1990.

Urban boundaries of four different years (1990, 1995, 2000 and 2005), which were derived from Landsat TM/ETM+ satellite data in this study, were overlaid on the UDSI map of Jingjinji area (Figure 5 -6 Beijing and Tianjin city for example), and the urban develop tendency in this area were indicated in Table 5.

It is found that the built-up areas of Beijing city have increased dramatically from 1990 to 2005. Meanwhile, Tian Jin city and Shi Jiazhuang city (the capital He Bei province) have higher average annual growth rates compared to other cities in this region. USDI information of each sub-region were calculated by overlapping vector city boundaries on the USDI map (Table 6).

According to the analysis results (Table 4 and Table 5) together with the urban planning policy in this area, it is found that the most suitable areas can meet the needs for future urban expansion within the coming 5-10 years. However, out of the total most suitable areas, about 60-80% were cropland. So that the future urban land use plan should be based on the utilization of limited the most suitable land together with the moderately suitable.

## 5. Conclusions

This paper focused on the integrated evaluation of urban development suitability for Jingjinji area (namely Beijing, Tianjin and He Bei Province) in North China, which is undergoing a process of rapid urban sprawl. Remote sensing and geographic information system (GIS) techniques were adopted, and a integrated simulating model was developed supported by an AHP method. The integrated urban development suitability index (UDSI) was calculated using this model. According to the UDSI result, the spatial distribution of urban development suitability and its driving forces were analyzed. The result of this study indicated that out of the total area of 21.36 km^2^, 22.64% (4.84 km^2^) are most suitable for urban development, 30.56% (6.53 km^2^) are moderately suitable for urban development and 46.81% (10.00 km^2^) of the area is least suitable for urban development.

The built-up areas of Beijing city has been increased dramatically from 1990 to 2005. Meanwhile, Tianjin city and Shi Jiazhuang city (the capital of He Bei province) have higher average annual growth rates compared to other cities in this region. According to the USDI information of each sub-region, it is found that the most suitable areas can meet the needs for future urban expansion within the coming 5-10 years. However, out of the total most suitable areas, about 60-80% were cropland. So any future urban land use plan should be based on the utilization of the most suitable land together with the moderately suitable.

The result of this study also indicated that integrated evaluation of urban development could be conducted in an operational way using remote sensing data, GIS spatial analysis technique and AHP modeling method.

## Supplementary Material



## Figures and Tables

**Table1.** Socio-economic information of Jingjinji area [15-17].

**Region**	**Area(km^2^)**	**Area of built-up land (km^2^, 2005)**	**Population (million capita, 2005)**	**GDP (billion RMB,2005)**
Beijing	16300	940.96	15.56	6814.5
Tianjin	11700	341.65	10.36	3663.86
He Bei	185600	1073.97	68.49	10116.6
Jingjinji area	213600	2356.58	94.40	20594.96

**Table 2.** Main factors and parameters for urban development suitability evaluation.

**Factors**	**Parameters**	**Scale**	**Data sources**
1 Environmental background	1.1 elevation	1:500,000	SBSC
	1.2 slope	1:500,000	SBSC
	1.3 geomorphological types	1:250,000	SBSC
	1.4 accumulated temperature (> 0°C)	1:100,000	RESDC
	1.5 wetness index	1:100,000	RESDC
2 Water/land resources	2.1 Precipitation	1:100,000	RESDC
	2.2 river density	1:100,000	RESDC
	2.3 land use	1:100,000	Derived from Landsat TM images
3 Socio-economic development	3.1 railway density	1:100,000	SBSC
	3.2 road density	1:100,000	SBSC
	3.3 population density	100m	RESDC

**Table 3.** The weighting matrix for urban development suitability evaluation

Factors	Weight	Parameters	Weight
1. Environmental background	0.10	1.1 elevation	0.25
		1.2 Slope	0.07
		1.3 geomorphological types	0.36
		1.4 accumulated temperature (> 0 °C)	0.11
		1.5 wetness index	0.21
2. Water/land resources	0.37	2.1 precipitation	0.22
		2.2 river density	0.11
		2.3 land-use types	0.67
3. Socio-economic development	0.53	3.1 railway density	0.25
		3.2 road density	0.13
		3.3 population density	0.62

**Table 4.** The standards for transforming original values of the parameters into relative scores

**Factors**	**Parameters**	**Standardized relative rates**		
		**1**	**3**	**5**
1 Environmental background	1.1 elevation	>4,000m	2,000-4,000m	<2,000m
	1.2 Slope	>15 degree	5-15 degree	<5 degree
	1.3 geomorphological types	abrupt mountain, sand hill	mountain, altiplano, mesa	hill, plain and all other types
	1.4 accumulated temperature (> 0 °C)	<500°C	500-1500°C	> 1500°C
	1.5 wetness index	>30%	10%-30%	<10%
2 water/land resources	2.1 Precipitation	<50 mm	50∼200 mm	>200 mm
	2.2 river density	<10	10∼100	>100
	2.3 land-use types	barren land, water	woodland, grass land	cultivate land, urban and rural settlements
3 socio-economic development	3.1 railway density	0	1-30	>31
	3.2 density	0	1-50	>51
	3.3 population density	0	1-100	>101

**Table 5.** Increasing of the built-up areas of the main cities in Jingjinji area (Unit: km^2^).

**Region**	**City**	**Built-up area (1990)**	**Built-up area (1995)**	**Built-up area (2000)**	**Built-up area (2005)**	**Average annual rate**
Beijing	Beijing	397.98	812.78	912.36	937.97	36.00
Tianjin	Tianjin	241.71	257.34	283.38	341.65	6.66
He Bei province	Tang Shan	86.36	97.91	114.4	118.11	2.12
	Qin Huangdao	47.5	56.58	64.34	79.59	2.14
	Lang Fang	19.3	44.12	46.74	49.09	1.99
	Shi Jiazhuang	86.32	130.8	150.03	162.69	5.09
	Bao Ding	44.25	66.43	77.26	78.39	2.28
	Cang Zhou	33.37	43.35	52.03	55.43	1.47
	Zhang Jiakou	28.12	34	35.09	39.16	0.74
	Chen De	14.18	15.47	17.97	19.05	0.32
	Xin Tai	31.53	34.6	41.18	44.03	0.83
	Han Dan	47.93	52.66	60.45	101.18	3.55
	Heng Shui	18.47	21.31	34.06	41.08	1.51
	Total province	457.33	597.23	693.55	787.8	2.00
Jingjinji area		1,097.02	1,667.35	1,889.29	2,067.42	4.98

**Table 6.** USDI information of each sub-region.

Sub-region	Most suitable (%)	Moderately suitable (%)	Least suitable (%)	Most suitable (without cropland, %)	Built-up areas in 2005 (%)
Beijing	20.65	39.10	40.26	6.73	5.75
Tianjin	25.77	25.77	48.46	2.96	2.84
He Bei province	22.56	30.13	47.31	9.51	0.57
