# Correction: Dong, J. *et al*. Integrated Evaluation of Urban Development Suitability Based on Remote Sensing and GIS Techniques–A Case Study in Jingjinji Area, China. *Sensors* 2008, *8*, 5975–5986

**DOI:** 10.3390/s100100933

**Published:** 2010-01-25

**Authors:** Jiang Dong, Dafang Zhuang, Xinliang Xu, Lei Ying

**Affiliations:** State Key Lab of Resources and Environmental Information System, Institute of Geographical Sciences and Natural Resources Research, Chinese Academy of Sciences, Beijing 100101, China

We found that formula (1) was incorrect in our paper published in *Sensors* in 2008 [1]. Therefore, formula (1) is corrected as follows:

(1) $$\text{UDSI}=\sum_{i=1}^{n}\text{Wi}*\text{Ci}$$
