# Supplementary material for: Integrated Evaluation of Urban Development Suitability Based on Remote Sensing and GIS Techniques – A Case Study in Jingjinji Area, China
Source: Sensors (Basel). 2008 Sep 25;8(9):5975–86. doi: 10.3390/s8095975 (PMC3705542; doi:10.3390/s8095975)
Supplement: Supplementary file 1 [file sensors-08-05975-s001.pdf]

Sensors **2010**, *10*, 933; doi:10.3390/s100100933

OPEN ACCESS

sensors

ISSN 1424-8220

www.mdpi.com/journal/sensors

Correction

Correction: Dong, J. *et al.* Integrated Evaluation of Urban Development Suitability Based on Remote Sensing and GIS Techniques–A Case Study in Jingjinji Area, China. *Sensors* 2008, *8*, 5975–5986

Jiang Dong *, Dafang Zhuang, Xinliang Xu and Lei Ying

State Key Lab of Resources and Environmental Information System, Institute of Geographical Sciences and Natural Resources Research, Chinese Academy of Sciences, Beijing 100101, China

* Author to whom correspondence should be addressed; E-Mail: jiangd@igsnrr.ac.cn; Tel. +86-10-64889433.

Received: 25 January 2010 / Published: 25 January 2010

We found that formula (1) was incorrect in our paper published in *Sensors* in 2008 [1]. Therefore, formula (1) is corrected as follows:

$$\mathrm{UDSI} = \sum_{i=1}^{n} \mathrm{Wi} * \mathrm{Ci} \qquad (1)$$

References

1. Dong, J.; Zhuang, D.; Xu, X.; Ying, L. Integrated Evaluation of Urban Development Suitability Based on Remote Sensing and GIS Techniques–A Case Study in Jingjinji Area, China. *Sensors* **2008**, *8*, 5975–5986.

© 2010 by the authors; licensee Molecular Diversity Preservation International, Basel, Switzerland. This article is an open-access article distributed under the terms and conditions of the Creative Commons Attribution license (http://creativecommons.org/licenses/by/3.0/).
